# Judicial review of algorithmic administrative systems legality evidence and remedies in the smart city state

**DOI:** 10.3389/frai.2026.1802986

**Published:** 2026-04-13

**Authors:** Khalid Saleem Ameen, Tavga Abbas Towfiq, Kawar Mohammed Mousa, Mahrokh Pooyanmehr

**Affiliations:** 1 Department of International Law, Near East University, Nicosia, North Cyprus, Türkiye; 2 Department of Business Administration, Near East University, Nicosia, North Cyprus, Türkiye; 3 Department of Architecture, Girne American University, Girne, North Cyprus, Türkiye

**Keywords:** administrative law, algorithmic governance, judicial review, public law, smart cities

## Abstract

Smart city programs are increasingly used by governments to manage public services through digital and automated systems, and this development is closely linked with questions of administrative power, fairness, and accountability. Existing public law doctrines in common law systems were shaped for human decision-making and have not been fully adapted to administrative action carried out through algorithmic systems, which creates a clear gap in current legal thinking. The research is motivated by the growing use of algorithmic systems as effective decision-makers in areas such as utilities, mobility, welfare, and digital identity, especially in contexts marked by limited state capacity and prolonged emergency conditions. The study aims to examine how judicial review should respond to algorithmic administrative systems so that legality, fairness, and accountability remain protected while legitimate administrative goals are still met. Methodologically, the article adopts a doctrinal and normative legal research design based on structured analysis of public law doctrine, relevant judicial and administrative materials, and governance instruments on automated decision-making. The focus is on developing a doctrinal framework that treats algorithmic systems as legally reviewable decision infrastructures rather than neutral technical tools. The study highlights the importance of reviewing not only final automated outcomes but also earlier design choices, including data use, system objectives, and oversight mechanisms. The research is important because it offers courts and lawmakers clearer legal tools to assess algorithmic administration, with particular attention to settings where oversight is weak and emergency powers risk becoming normalized, increasing the danger of opacity, discrimination, and unchecked security repurposing.

## 1 Introduction

The increasing use of algorithmic systems in public administration has become a defining feature of contemporary governance, particularly within smart city programs that rely on digital infrastructures to manage public services and administrative functions (Meijer and Bolívar, 2016; Brauneis and Goodman, 2018). Governments across jurisdictions are adopting automated systems to support decision-making in areas such as welfare allocation, urban mobility, energy management, and digital identity, raising complex questions about legality and administrative accountability (Coglianese and Lehr, 2019; Tikhaleva, 2023; Shwan et al., 2025).

Administrative law has traditionally been structured around human discretion and reasoned judgment, yet the shift toward automated administrative action places pressure on long-standing legal doctrines developed for analog decision processes (Harlow, 2005; Koch, 1986). Judicial review, as a central mechanism for supervising administrative power, faces growing difficulty when decisions are generated or shaped by opaque computational processes rather than identifiable public officials (Cobbe, 2019; Williams, 2022).

Legal scholarship has increasingly examined algorithmic decision-making in the public sector, with attention given to how automation affects discretionary power, procedural fairness, and the duty to give reasons (Oswald, 2018; Civitarese Matteucci, 2021). Studies have shown that algorithm-assisted administration often blurs the boundary between rule-based decision-making and discretion, which complicates established standards of review under public law (Sjöberg, 2023; Mccann and Allard, 2023).

Concerns about transparency and accountability remain central in the literature, as algorithmic systems frequently rely on complex data processing and statistical models that resist conventional evidentiary scrutiny (Engstrom and Ho, 2020; Tomlinson et al., 2020). Scholars have argued that without access to intelligible explanations and audit records, courts may struggle to assess whether administrative decisions meet legality and rationality standards (Chauhan, 2020; Gontarz, 2023).

The rise of smart cities has intensified these challenges, as algorithmic governance becomes embedded within urban infrastructure and service delivery systems (Meijer and Bolívar, 2016; König, 2021). Research on smart urban governance highlights that data-driven platforms increasingly function as de facto administrative authorities, reshaping citizen and state relations in ways that test public law safeguards (Brauneis and Goodman, 2018; Bracci, 2023; Ul Mansoor, 2026).

Another significant body of literature addresses algorithmic transparency and disclosure duties, particularly in relation to the evidentiary demands of judicial review (Coglianese and Lehr, 2019; Cobbe and Singh, 2020). While transparency obligations are widely discussed, scholars note persistent uncertainty about how much technical detail courts should require and how such information can be made meaningful in legal proceedings (Sheehy and Ng, 2024; Ranchordás, 2024).

Procedural fairness has also been re-examined in the context of automated administration, especially where individuals are affected by high-stakes decisions without effective opportunities for challenge or human intervention (Sela, 2016; Czarnocki and Pałka, 2024). Existing administrative procedures often fail to account for the cumulative effects of data errors, model design, and threshold settings that shape automated outcomes (Ng et al., 2020; Haim, 2023).

Issues of discrimination and equality before the law occupy a growing place in discussions of algorithmic administration, as data-driven systems have been shown to reproduce or intensify unequal treatment across protected groups (Gerards and Zuiderveen Borgesius, 2020; Zuiderveen Borgesius, 2020). Legal scholars have questioned whether existing non-discrimination frameworks are adequate to address indirect bias embedded within algorithmic decision pathways (Kelly-Lyth, 2023; Kumkumoğlu and Kumkumoğlu, 2024).

Recent work has examined evidentiary burdens in proving algorithmic discrimination, pointing to difficulties faced by claimants when access to model logic and performance data is limited (Grozdanovski, 2021). Courts are often required to assess fairness without full visibility into how automated systems classify, rank, or exclude individuals (Weerts et al., 2023; Cromvelle, 2025).

Despite this growing body of scholarship, much of the literature remains fragmented across debates on transparency, discretion, fairness, and discrimination, without offering a unified doctrinal structure for judicial review of algorithmic administration. Existing studies often address isolated aspects of automated decision-making, while leaving unanswered how courts should systematically assess legality, evidence, and remedies across the full lifecycle of algorithmic systems (Movahed et al., 2026).

There remains a lack of clear public law guidance on how judicial review should engage with upstream system design choices, ongoing system operation, and the repurposing of civic data infrastructures within smart city governance. The absence of such a framework is especially concerning in contexts marked by institutional fragility and prolonged emergency conditions, where automated systems may operate with limited oversight (Simmler et al., 2023).

The aim of this study is to examine how judicial review can be structured to assess algorithmic administrative systems in a manner that preserves legality, fairness, and accountability. Methodologically, the article proceeds through doctrinal and normative legal analysis of scholarship, selected judicial and administrative materials, and official governance instruments relevant to automated decision-making. The study seeks to develop a doctrinal framework that treats algorithmic systems as legally reviewable administrative structures rather than neutral technical instruments, with attention to evidence standards and judicial remedies suited to automated governance (Shan, 2026; Gürler et al., 2024).

## 2 Research design and methodology

This study is designed as a doctrinal and normative legal analysis rather than an empirical investigation. Doctrinal legal research is appropriate where the objective is to identify the legal principles, categories, and review standards that should govern a developing field of public decision-making, while normative analysis is necessary where existing doctrine remains incomplete and requires structured legal reconstruction (Byrne and Olsen, 2024; De Witte, 2022). The article therefore asks how common law judicial review can be adapted to algorithmic administrative systems without abandoning core public law requirements of legality, fairness, relevance, transparency, and accountability.

Source selection was guided by direct relevance to administrative decision-making and the judicial control of public power. Priority was given to four categories of material: scholarship on administrative law and algorithmic governance; scholarship on smart cities and digital government where automated systems mediate public functions; official governance instruments for automated decision-making in the public sector; and illustrative judicial and administrative materials that expose practical legal problems in automated governance. The official instruments consulted include Canada’s published guidance and impact-assessment materials on automated decision-making, the United Kingdom’s Algorithmic Transparency Recording Standard, the NIST AI Risk Management Framework, and the European Union’s AI Act, because these instruments specify concrete expectations concerning documentation, oversight, transparency, record-keeping, and risk management in automated decision-making (Custers and Heijne, 2022; Krook et al., 2025; Wasil et al., 2025; Smuha, 2025). Illustrative judicial and administrative materials were also used to keep the analysis connected to legal practice, including the SyRI litigation in the Netherlands, the CJEU’s judgment in SCHUFA Holding (Scoring), and the Australian Robodebt inquiry (Rachovitsa and Johann, 2022; Priergaard, 2025).

**Figure 1:**
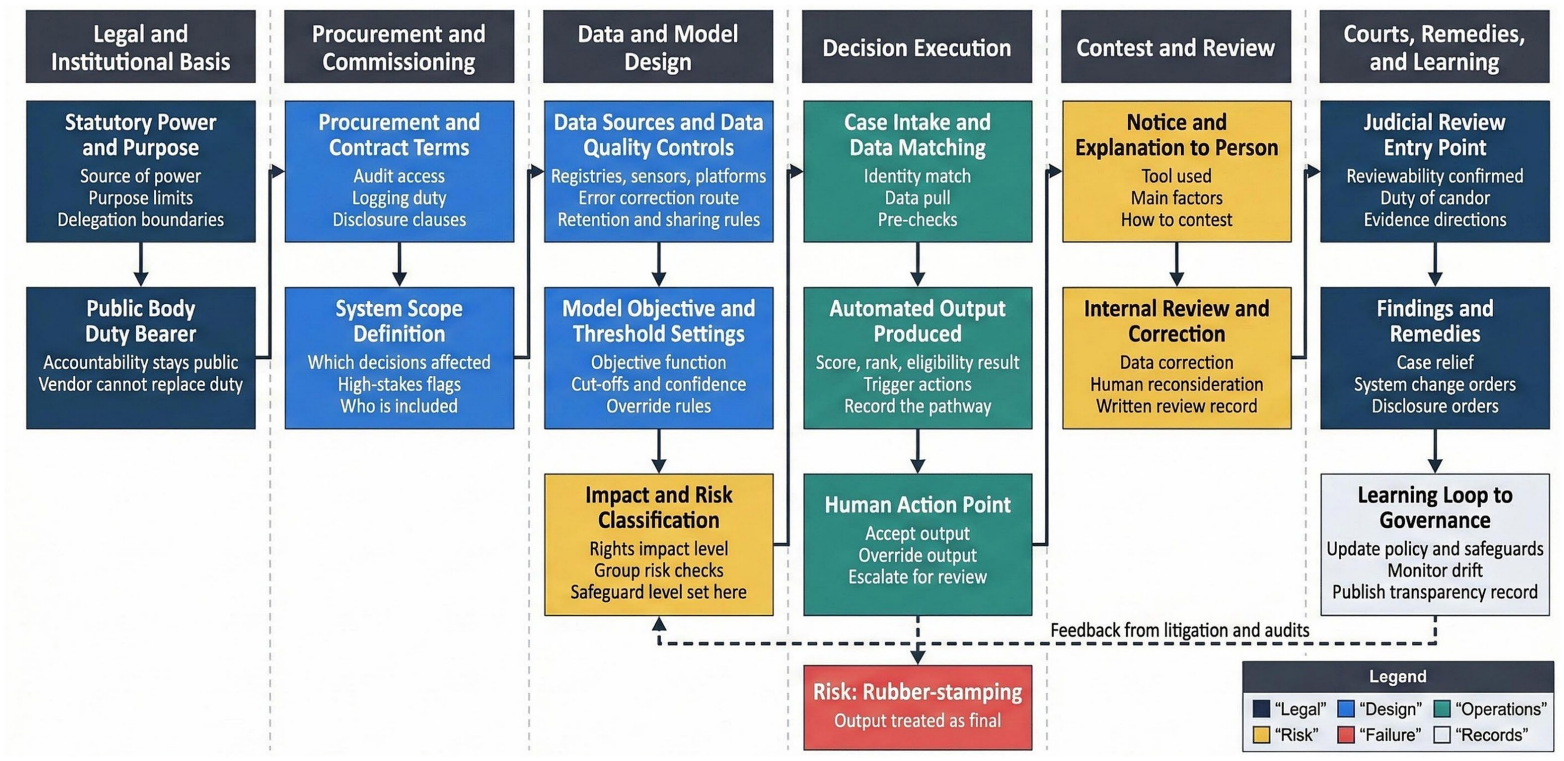
Algorithmic administrative systems as decision infrastructures, showing the full chain from legal authority to outputs and review.

**Figure 2:**
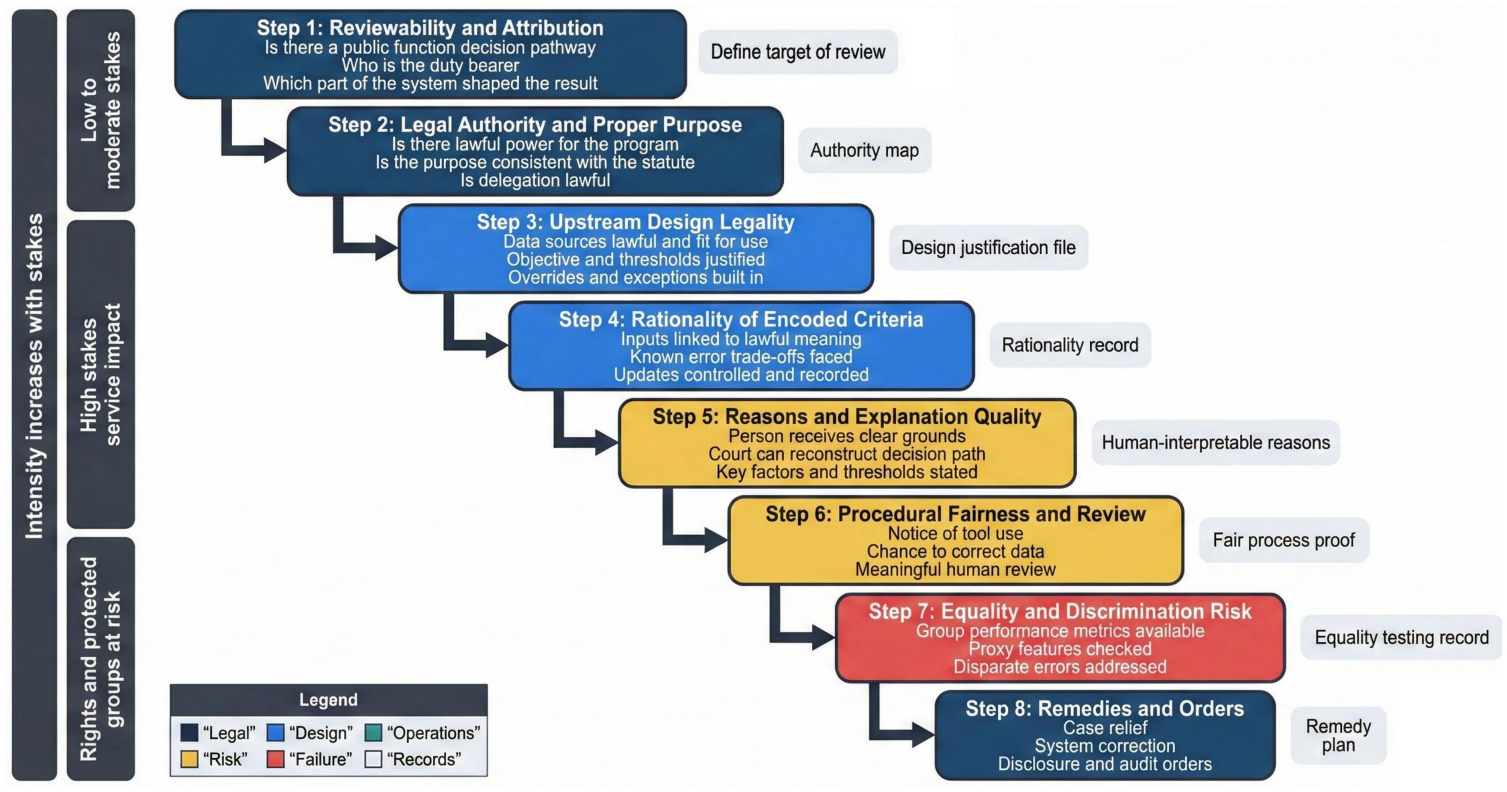
Structured judicial review questions applied in sequence, from authority to reasons, and equality checks.

**Figure 3:**
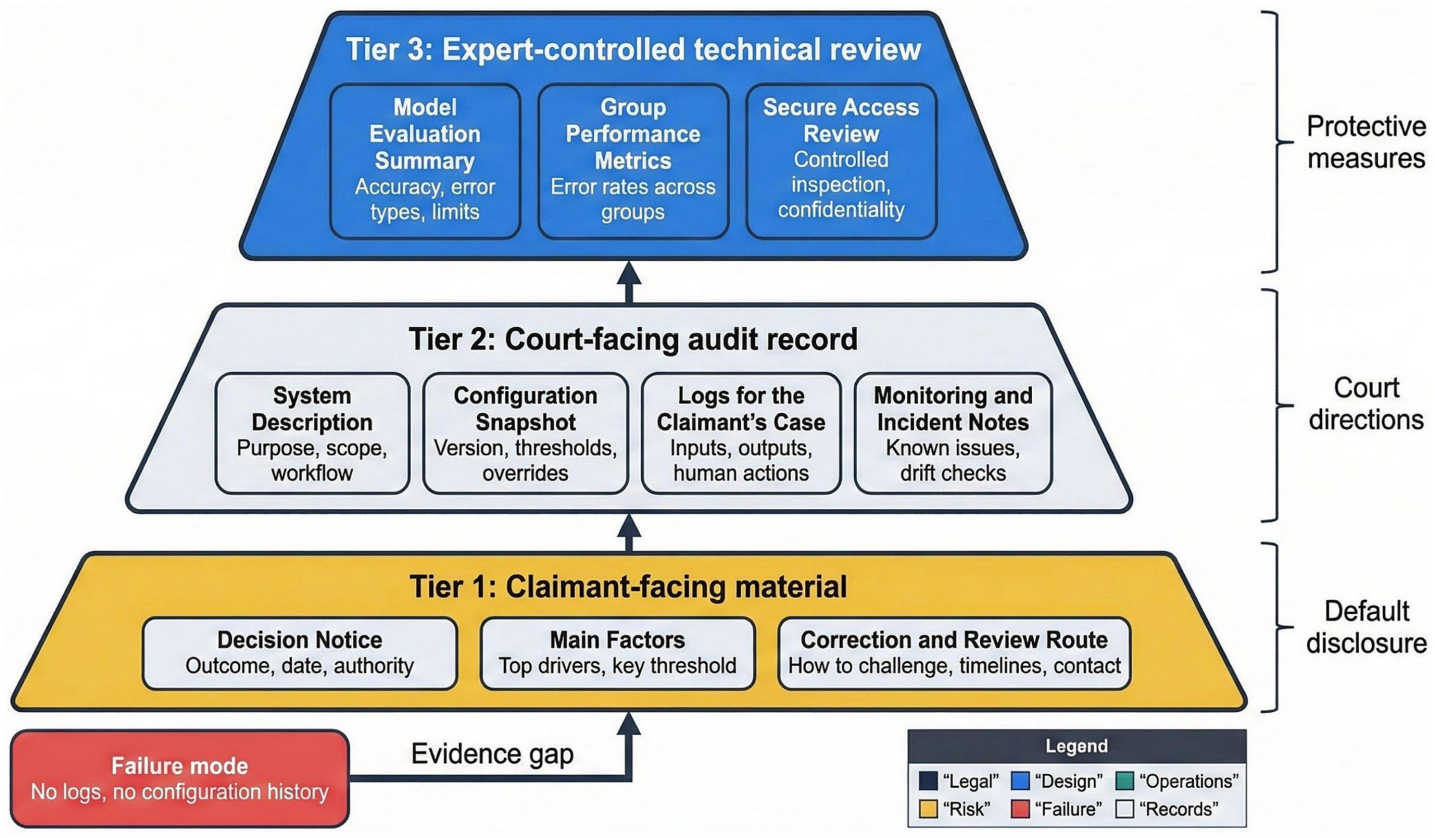
Tiered disclosure and evidence structure, showing what is provided to the person, to the court, and to experts.

**Figure 4:**
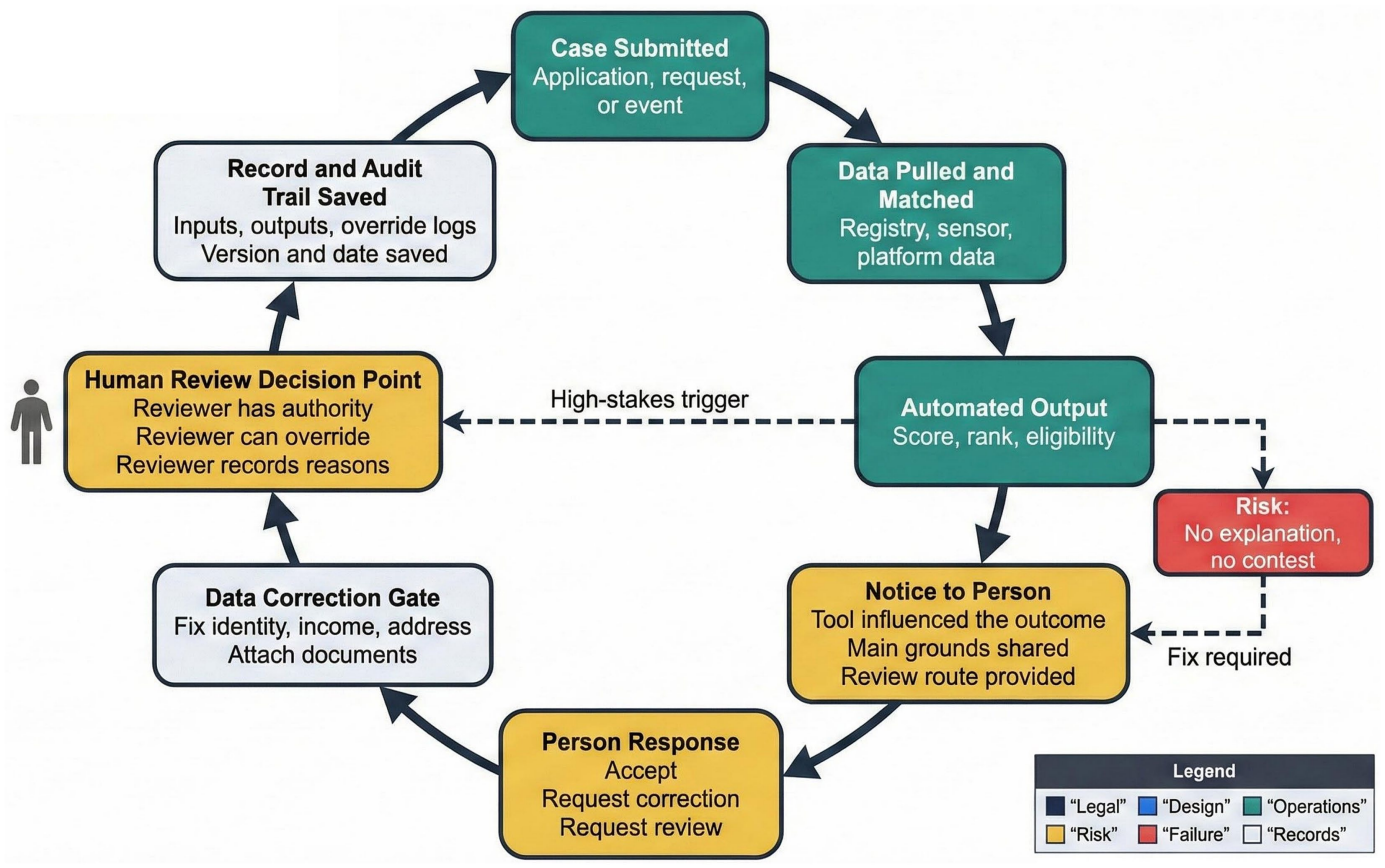
Procedural fairness architecture, showing notice, contest, correction, and genuine human review points.

**Figure 5:**
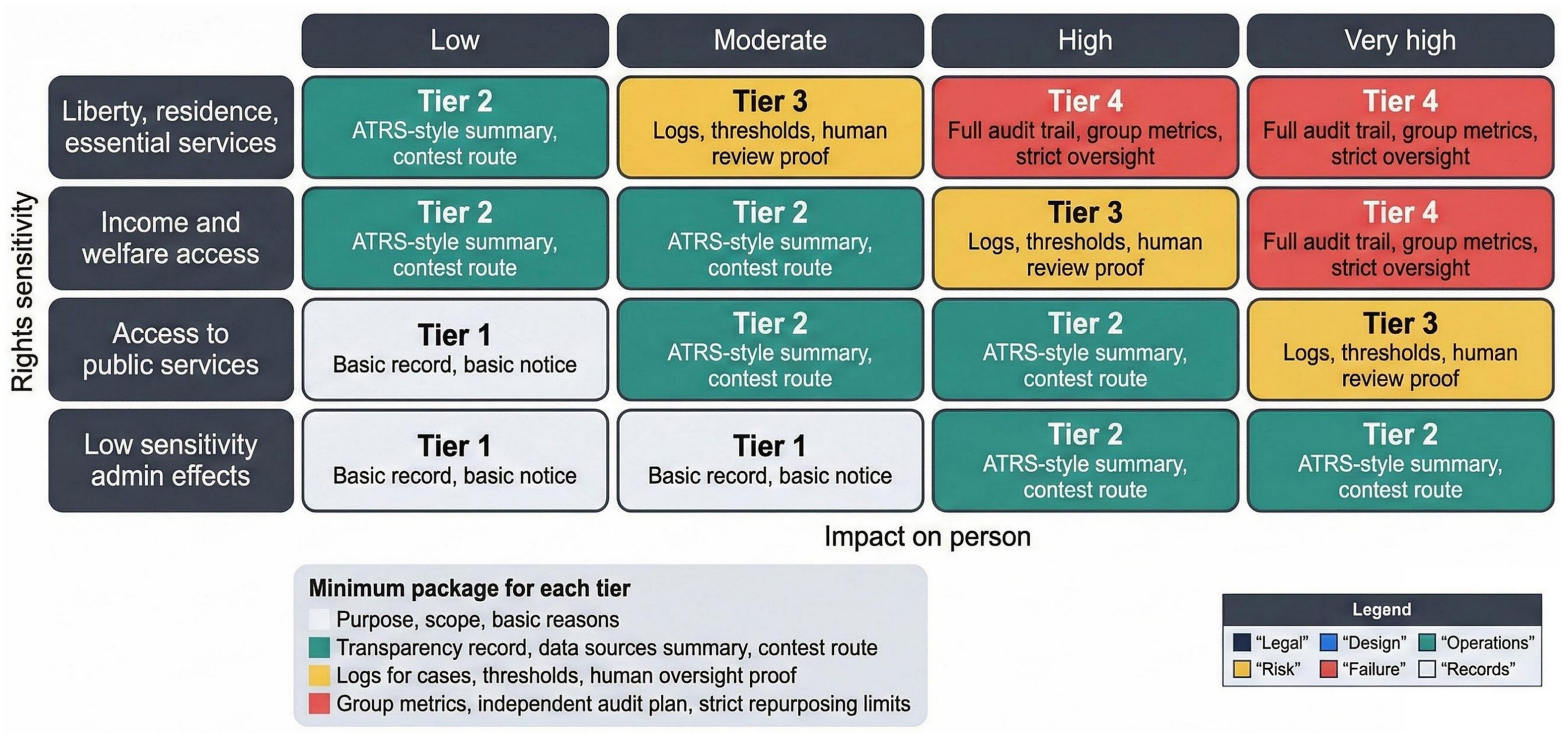
Risk tier matrix for algorithmic public administration, mapping impact level to review intensity and evidence expectations.

**Figure 6:**
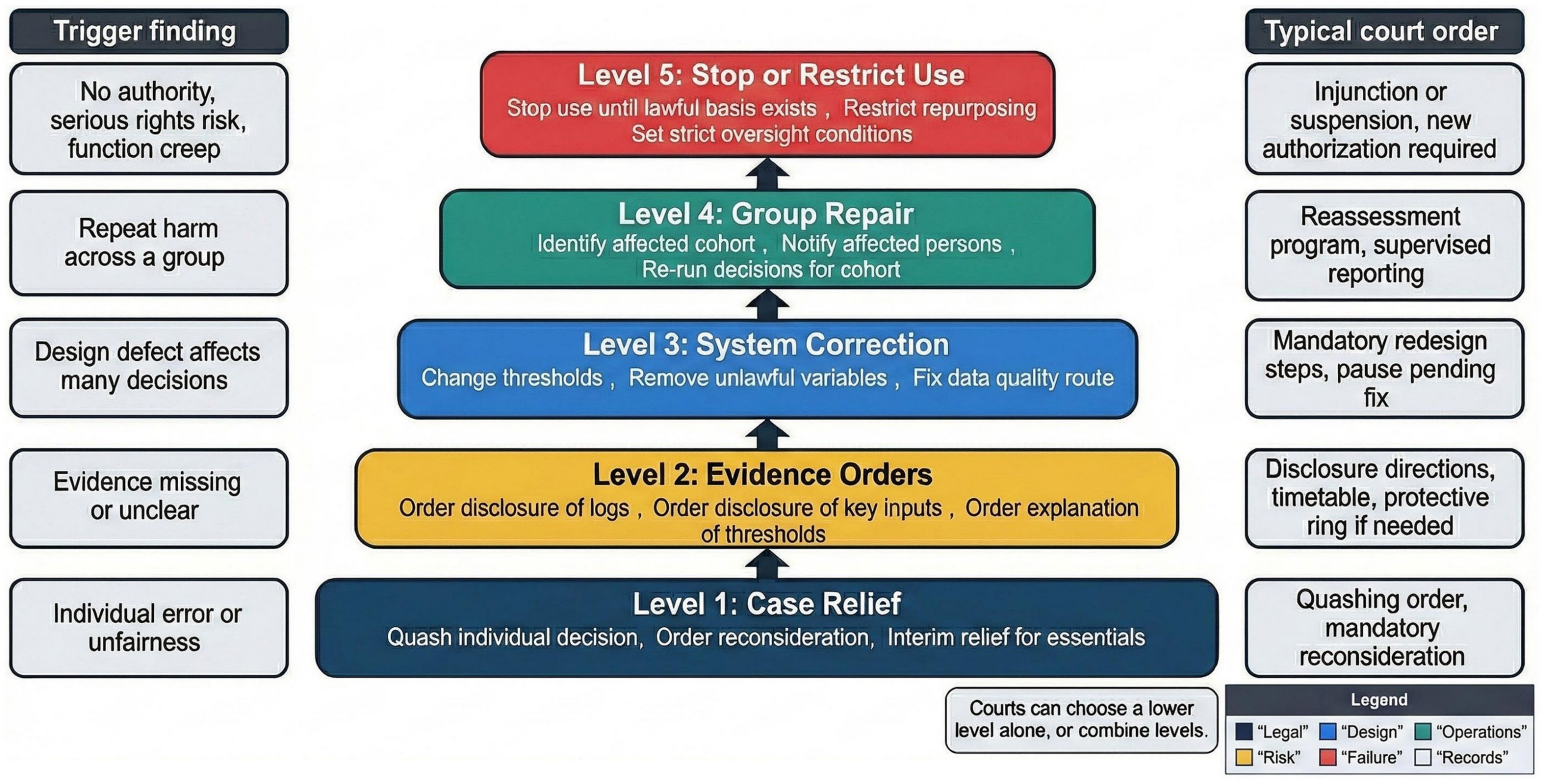
Remedy ladder for unlawful algorithmic administration, from individual correction to program-wide repair.

**Figure 7:**
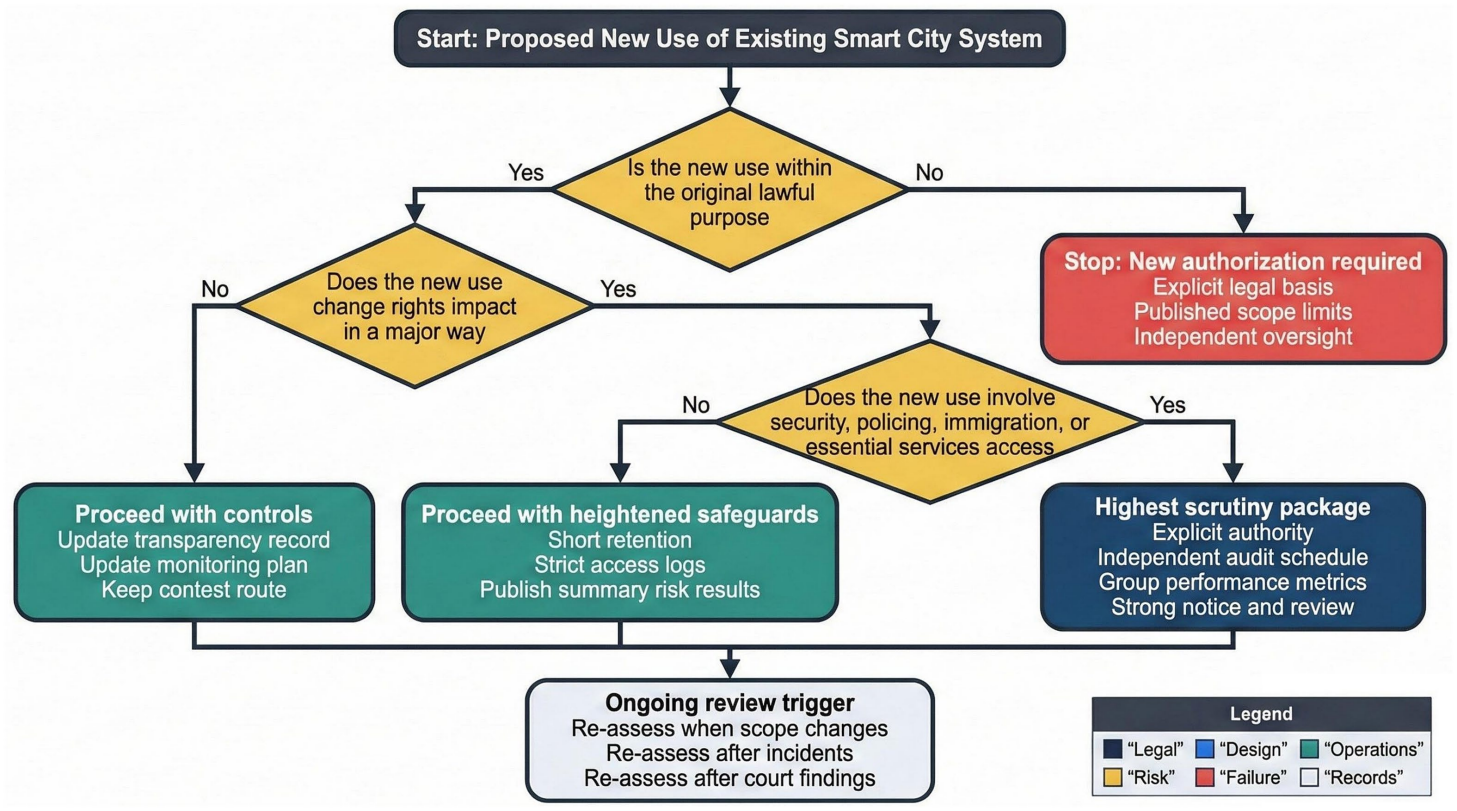
Bounded function creep test for repurposing civic datasets or algorithmic functions into security-related use.

The analytical procedure followed four steps. First, the study identified recurring legal problems in the literature, particularly those relating to authority, discretion, procedural fairness, reasons, evidence, discrimination, and remedies. Second, it examined how those problems appear in selected judicial and administrative materials and in public-sector governance instruments for automated decision-making. Third, it organized the recurring issues through established public law concepts, including legality, proper purpose, relevant considerations, fettering, reason-giving, equality, and remedial proportionality. Fourth, it translated that synthesis into the framework presented in Tables 1–5 and Figures 1–7. These tables and figures are therefore analytical syntheses derived from doctrinal review; they are not presented as empirically validated models or as settled positive law across all jurisdictions.

**Table 1:** Reviewable decision infrastructure map for legality, evidence, and remedies.

Layer of inquiry	Main public law question	What the court needs to see	Typical legal risk	Likely forms of relief
Reviewability and attribution	Is there a reviewable public law act, and who is responsible?	System description, governance chain, vendor role, delegation terms	“Black box” outsourcing, unclear duty bearer	Declaration of responsibility, directions for evidence, joinder or disclosure orders
Legal authority and proper purpose	Is the system authorized, and is its purpose lawful?	Statutory basis, policy basis, data-sharing authority, procurement constraints	Ultra vires, improper purpose, unlawful delegation, fettering	Quashing, declaration, suspended quashing where needed
Design legality	Are upstream choices lawful and consistent with public law duties?	Data specification, model objective, thresholds, override rules, change control	Built-in bias, proxy discrimination, unlawful criteria	Mandatory order to redesign, disclosure, re-run determinations
Decision legality and fairness	Did the specific decision meet legality, rationality, and fairness?	Individual record, reasons, contest route, human review record	No meaningful reasons, no participation, rubber-stamping	Quashing of decision, mandatory reconsideration, interim relief
Evidence and remedies for system-wide harm	Can the court test legality and craft an effective remedy?	Logs, audits, performance metrics by group, impact assessments	Proof barriers, repeat harm, secrecy claims	Structured remedies, ongoing compliance reporting, targeted disclosure

**Table 2:** Minimum record for review in algorithmic administrative systems.

Record item the court can require	Why it is legally relevant	Where it appears as a baseline expectation
Statutory or lawful policy basis for automation choice	Authority, purpose, relevance	ATRS “why used” fields
System scope, decision points, and where outputs bind practice	Reviewability, fettering, discretion location	Canada scope guidance
Data sources, feature list, and exclusion rules	Legality of criteria, proxy bias, relevance	AIA risk questions focus on data and impacts
Model objective, thresholds, and error trade-offs	Rationality, proportionality-style reasoning, equality risk	Model documentation norms
Human oversight design, override rules, escalation routes	Fairness, meaningful review, accountability	EU AI Act high-risk focus on oversight
Logging and audit trail design	Evidence production, duty of candor, repeat harm detection	EU AI Act record-keeping duties
Monitoring plan and change control	Ongoing legality, drift, unlawful continuation	NIST AI RMF governance focus
Equality and discrimination testing plan	Non-discrimination duties, proof readiness	EU AI Act and model card testing logic
Vendor terms for audit and disclosure	Accountability in outsourced tools	Transparency governance logic

**Table 3:** Evidence and disclosure matrix for algorithmic judicial review.

Category of material	Default audience	Reason
Individual factors and main grounds for decision	Claimant	Participation and contest route
Data quality issues affecting the claimant	Claimant	Ability to correct errors
System description and purpose statement	Public	Democratic accountability, legality check
Governance record, impact assessment summary	Public, with redactions where justified	Public justification for high-impact tools
Technical documentation, parameter ranges, change control	Court and experts under protection	Testing legality without broad exposure
Logs and audit trails for the claimant’s case	Court and experts, partial claimant access	Reconstructing decision path
Performance metrics across groups	Court and experts, publish summaries where safe	Discrimination testing and legality
Security-sensitive operational details	Court only, tightly controlled	Preventing misuse while allowing legality review

**Table 4:** Remedies menu for unlawful algorithmic administration.

Type of finding	Typical form of illegality	Remedies that fit the harm
Individual decision error	Wrong inputs, wrong application of rules, unfair process	Quash decision, mandatory reconsideration, interim relief
Design illegality	Unlawful criteria, unlawful thresholds, proxy discrimination	Declaration of unlawfulness, mandatory redesign order, temporary stop on use
Evidence failure	No logs, no governance record, non-candor	Mandatory disclosure, directions for audit trail creation, adverse inference where justified
Ongoing illegality	Continued use despite known defect	Declaration, mandatory suspension until fixes, reporting obligations
Group harm	Unequal error patterns, disparate impact	Order for metrics disclosure, targeted re-run for affected cohorts, compliance monitoring
Function repurposing without authority	Civic data reused for security aims	Declaration, stop order, requirement for explicit legal basis

**Table 5:** Bounded function creep test for smart city systems.

Step	Question	Evidence needed
1	What was the original lawful purpose and legal basis?	Statute, policy, ATRS record
2	What is the new purpose, and is it materially different?	Repurposing decision record, updated impact assessment
3	Is there explicit authority for the new purpose?	Legislative text, delegated powers, safeguards
4	What limits exist on scope, retention, sharing, and access?	Data governance rules, retention schedule, access logs
5	What rights and group risks are triggered?	Equality metrics, risk analysis, contest route
6	What independent oversight exists?	Audit plan, external review, reporting

## 3 Literature review

### 3.1 Smart cities and digital government in public law scholarship

Smart city governance is widely described as a shift in how public authority is organized, because many administrative functions are carried through connected infrastructures, data platforms, and automated service systems (Meijer and Bolívar, 2016; Tikhaleva, 2023). Legal writing on smart cities often links these technologies with questions about public power, public accountability, and legal authorization, since service delivery is increasingly mediated through data collection and computational systems (Brauneis and Goodman, 2018; König, 2021). Discussions of smart urban governance also emphasize that digital government reforms are rarely neutral in their legal effects, because system design choices can reshape access to rights and public benefits through everyday administrative routines (Meijer and Bolívar, 2016; Mastrogiovanni, 2025; Nabawagga et al., 2026).

Research on algorithmic transparency in smart cities highlights that public law oversight becomes harder when decisions and resource allocations are produced through systems that ordinary users cannot inspect or understand (Brauneis and Goodman, 2018; Coglianese and Lehr, 2019). Studies on citizen-centered data governance argue that legitimacy in smart city management depends on governance structures that can justify data collection, data sharing, and automated action under clear accountability conditions (König, 2021; Mastrogiovanni, 2025). Accounting and governance scholarship further argues that “intelligent” public services may create gaps in responsibility, especially when multiple contractors, data providers, and agencies jointly shape how decisions occur (Bracci, 2023; Anshari et al., 2025).

Public law scholarship on the digital state frames these issues through rights protection and administrative justice, with emphasis on how transparency and review pathways may weaken when systems are complex and layered (Ng et al., 2020; Ranchordás, 2024). Authors also argue that smart city governance can expand executive capacity without matching legal checks, particularly where digital reforms are introduced through policy programs rather than legislation (Tikhaleva, 2023; Ng et al., 2020). Table 6 summarizes how the smart city and digital government literature connects governance structures with legal issues such as authorization, accountability, and reviewability, and it shows how these themes connect to administrative law concerns about legality and fairness (Meijer and Bolívar, 2016; Brauneis and Goodman, 2018).

**Table 6:** Smart city and digital government themes in public law and governance scholarship.

Theme area	Main focus	Linked legal concerns	Representative references
Smart urban governance models	Governance arrangements for data-driven city management	Democratic authorization, accountability, allocation power	Meijer and Bolívar (2016) and Tikhaleva (2023)
Smart city transparency	Visibility of decision processes and infrastructure decisions	Reviewability, access to reasons, contestability	Brauneis and Goodman (2018) and Coglianese and Lehr (2019)
Citizen-centered data governance	Accountability for data collection, sharing, and use	Legality of processing, limits on repurposing, legitimacy	König (2021) and Mastrogiovanni (2025)
“Intelligent” public services and accountability gaps	Fragmented responsibility across agencies and vendors	Attribution of legal responsibility, audit duties	Bracci (2023) and Anshari et al. (2025)
Digital-era public law renewal	Rights, transparency, administrative justice in technology settings	Procedural protection, access to review, fairness duties	Ng et al. (2020) and Ranchordás (2024)
Algorithmic governance transparency	Disclosure and explanation duties for algorithmic administration	Evidentiary access, rationality review, reasons-giving	Coglianese and Lehr (2019) and Engstrom and Ho (2020)

### 3.2 Algorithmic decision-making and administrative discretion

Administrative discretion has long been a central concept in public law, because it describes the space where officials choose between lawful options and balance competing public purposes (Koch, 1986; McHarg, 2017). Automated decision-making challenges this concept, since discretion may appear to shift from frontline officials to system designers, data choices, and model objectives that structure the decision process before any individual case is assessed (Oswald, 2018; Mccann and Allard, 2023). Scholarship on automated public-sector decisions argues that algorithmic systems can become administrative “machines of government,” where discretion is exercised through design and configuration rather than a human reasoning process recorded in the file (Cobbe, 2019; Civitarese Matteucci, 2021).

Studies that examine administrative law and automation argue that traditional categories, such as rules versus discretion, become less stable when systems combine rigid thresholds with probabilistic scoring and risk rankings (Oswald, 2018; Williams, 2022). Work on the rule of law in algorithmic administration connects this to legality concerns, since system outputs can carry the force of administrative authority even when the internal basis for a decision is difficult to explain in legal terms (Civitarese Matteucci, 2021; Haim, 2023). A related discussion focuses on the internal governance of administrative algorithms, suggesting that legal controls should address the internal “law” of the system, such as documentation, model governance, audit trails, and internal review routes (Haim, 2023; Cobbe and Singh, 2020).

Regulatory design proposals argue that government adoption of AI should be matched with institutional design features that preserve administrative responsibility and rights-based safeguards (Sheehy and Ng, 2024; Sawicki, 2025). Comparative accounts also suggest that administrative courts may face specific difficulty in identifying “unlawful regimes” where the unlawfulness is produced by system settings or data pipelines rather than a visible official act (Gontarz, 2023; Daly et al., 2023). Table 7 maps how the discretion literature identifies different “locations” of discretion in algorithmic administration, including upstream design, operational deployment, and human override practices, and links these locations to review challenges under administrative law (McHarg, 2017; Mccann and Allard, 2023).

**Table 7:** Locations of discretion and review challenges in algorithmic administrative systems.

Location where discretion operates	How discretion appears in algorithmic administration	Why judicial review becomes difficult	Representative references
Legislative and rule design stage	Broad statutory goals leave room for design choices	Courts may face deference and limited record visibility	Koch (1986) and McHarg (2017)
System design and configuration	Objectives, thresholds, override rules shape outcomes	Unlawfulness may arise before any single decision	Oswald (2018) and Haim (2023)
Data selection and data quality	Training data and features influence classifications	Claimants may lack access to data pipelines	Cobbe and Singh (2020) and Wu (2023)
Operational deployment in agencies	Automated outputs guide or replace human judgment	Record-keeping may not show legal reasoning steps	Cobbe (2019) and Tomlinson et al. (2020)
Human oversight and exception handling	Officials accept, override, or rubber-stamp outputs	Accountability may shift and become unclear	Mccann and Allard (2023) and Cobbe and Singh (2021)
Court-facing evidence production	Agencies present summaries instead of system details	Courts may not test legality without auditable pathways	Engstrom and Ho (2020) and Sjöberg (2023)
Equality assessment and discrimination proof	Performance differs across protected groups	Proof burdens and causation are hard without metrics	Gerards and Zuiderveen Borgesius (2020) and Grozdanovski (2021)
Remedy design after unlawfulness	System-wide fixes, disclosure orders, re-run decisions	Traditional remedies focus on single decisions	Cromvelle (2025) and Zuiderveen Borgesius (2020)
Procedural fairness and participation	Notice, reasons, and contest rights become weaker	Affected persons may not understand decision basis	Sela (2016) and Czarnocki and Pałka (2024)
Regulatory design for government AI	Institutional design for lawful adoption	Lack of agreed standards for governance duties	Sheehy and Ng (2024) and Sawicki (2025)

### 3.3 Judicial review, legality, and procedural fairness in automated systems

Judicial review is often framed as the core tool for supervising administrative legality, yet automated systems add evidentiary and doctrinal strain because courts may not easily trace how a decision was made or which legal reasons supported it (Cobbe, 2019; Tomlinson et al., 2020). Scholarship on reviewing automated administrative acts argues that courts face a threshold problem of how to characterize algorithmic outputs, whether as reviewable “decisions,” as parts of a broader administrative process, or as technical steps outside public law control (Daly et al., 2023; Sjöberg, 2023). This concern is connected to calls for rethinking administrative law for algorithmic decision-making, where the standard ideas of error, irrationality, and relevant considerations may require adaptation to system-based governance (Williams, 2022; Chauhan, 2020).

Procedural fairness is also a recurring theme, especially where affected individuals cannot understand the basis for a decision or cannot meaningfully challenge it (Cobbe and Singh, 2021; Ng et al., 2020). Work on procedural justice in automated dispute resolution suggests that perceptions of fairness depend on intelligible processes and real opportunities to participate, even when a system is technically accurate (Czarnocki and Pałka, 2024). Studies focusing on due process in automated public services argue that digital administration needs procedural rules that fit automated workflows, including notice standards, review rights, and human involvement for high-stakes determinations (Ranchordás, 2024; Cobbe and Singh, 2021).

Evidence in judicial review is discussed as a separate pressure point, since conventional judicial review evidence often relies on written reasons and administrative records, while algorithmic systems may generate outputs without comparable records that show legal reasoning (Tomlinson et al., 2020; Engstrom and Ho, 2020). Authors also link this to proceduralism concerns, warning that automated administration may weaken the values of openness and accountability if courts accept minimal explanations or accept technical opacity as unavoidable (Harlow, 2005; Cobbe, 2019). More recent writing argues that administrative courts may need structured methods for assessing legality and reviewing system governance, especially when unlawfulness may arise from design rather than an isolated decision error (Gontarz, 2023; Sjöberg, 2023).

### 3.4 Accountability, transparency, and non-discrimination in algorithmic governance

Accountability and transparency are central themes in the literature on algorithmic governance, with many authors arguing that legal oversight requires access to meaningful information about how decisions are produced (Coglianese and Lehr, 2019; Engstrom and Ho, 2020). Scholarship on algorithmic public services argues that accountability gaps can arise when agencies rely on vendor tools, complex data chains, or automated models that distribute responsibility across actors (Bracci, 2023; Anshari et al., 2025). Proposals for “reviewable” automated decision-making suggest that accountability should be grounded in documentation, system logs, traceable decision pathways, and clear duties assigned to public bodies, even when external tools are involved (Cobbe and Singh, 2020; Sheehy and Ng, 2024).

Transparency debates in smart city contexts often highlight the difference between formal disclosure and meaningful explanation, since technical disclosures may not help courts or affected persons understand why a result occurred (Brauneis and Goodman, 2018; Coglianese and Lehr, 2019). Work on data governance in smart cities connects this issue with public trust, privacy, and legitimacy, arguing that governance models need stronger legal authorization and accountability arrangements for data processing and repurposing (König, 2021; Mastrogiovanni, 2025). Public law scholarship in the technological era links transparency to rights-based review, suggesting that administrative justice can weaken when systems remain opaque or when access to reasons becomes limited (Ng et al., 2020; Ranchordás, 2024).

Non-discrimination law has become a major focus because algorithmic systems can generate biased outcomes through data patterns, proxy variables, and unequal error rates across protected groups (Zuiderveen Borgesius, 2020; Gerards and Zuiderveen Borgesius, 2020). Labor and equality scholarship highlights that algorithmic discrimination can occur even without intent, which creates legal questions about proof, causation, and appropriate remedies (Kelly-Lyth, 2023; Cromvelle, 2025). EU-focused work argues that existing legal categories do not map cleanly onto how models operate, since the law often expects structured decision logic while algorithmic systems operate through statistical correlations and complex feature spaces (Weerts et al., 2023; Grozdanovski, 2021; Fraenkel-Haeberle, 2026). Scholarship also discusses how new regulatory measures and digital equality trends attempt to operationalize non-discrimination duties in technology governance, while still leaving open questions about enforcement and litigation strategy (Hacker et al., 2024; Kumkumoğlu and Kumkumoğlu, 2024).

### 3.5 Limitations and unresolved questions in existing legal literature

The existing literature provides strong building blocks on legality, fairness, transparency, and discrimination, yet several unresolved issues remain in how these strands connect into a coherent judicial review approach for algorithmic administration (Williams, 2022; Cobbe, 2019). Some work addresses judicial review doctrine directly, yet it may focus on review of individual decisions without fully addressing how courts should treat upstream design decisions, data governance choices, and system objectives as part of legality analysis (Sjöberg, 2023; Haim, 2023). Other work stresses systemic review, but it often leaves open how such review fits within conventional judicial review procedure, evidentiary limits, and standards of deference (Chauhan, 2020; Tomlinson et al., 2020).

Comparative and institutional scholarship indicates that administrative courts may face difficulty in diagnosing unlawfulness where the fault lies in data quality, model thresholds, or performance differences across groups, especially when technical access is contested (Gontarz, 2023; Engstrom and Ho, 2020). Transparency proposals frequently argue for disclosure duties and explanation requirements, yet there remains disagreement about what level of technical detail is enough for legal control and what forms of explanation support fair participation (Coglianese and Lehr, 2019; Brauneis and Goodman, 2018). Procedural fairness scholarship points to the need for human review and participation rights in high-stakes settings, yet the literature still lacks agreement on minimum standards for meaningful oversight when systems are embedded across agencies and platforms (Czarnocki and Pałka, 2024; Ranchordás, 2024).

Non-discrimination scholarship identifies large proof barriers, especially when claimants cannot access performance metrics or training data that could show unequal error patterns (Grozdanovski, 2021; Wu, 2023). Even where discrimination is established, legal scholarship continues to debate what remedies should look like when harm stems from a system’s design and repeated deployment rather than a single unlawful act (Cromvelle, 2025; Zuiderveen Borgesius, 2020).

Emerging judicial and administrative practice already illustrates the types of problems identified in the literature. In the SyRI case, the Hague District Court held that the Dutch legislative framework for automated fraud-risk detection failed to comply with Article 8 ECHR because the operation of the system was insufficiently transparent and verifiable, showing how opacity can itself become a legality defect in public administration (Rachovitsa and Johann, 2022). In SCHUFA Holding (Scoring), the Court of Justice of the European Union held that automated scoring may fall within the prohibition on automated individual decision-making where third parties rely strongly on that score, underscoring the legal importance of explanation, access, and the downstream effects of automated classification (Custers and Heijne, 2022). The Australian Robodebt inquiry likewise revealed how automated debt generation combined with weak human checking and poor legal oversight can produce repeated administrative unlawfulness at scale (Priergaard, 2025). These materials do not amount to a full comparative case study, but they provide concrete jurisprudential and administrative anchors for the framework developed in this article.

## 4 Proposed framework for judicial review of algorithmic administrative systems

### 4.1 Conceptualizing algorithmic systems as reviewable decision infrastructures

Algorithmic systems used in public administration increasingly operate as structured decision pathways rather than isolated technical tools, and this functional role requires a corresponding legal understanding within judicial review doctrine (Cobbe, 2019; Williams, 2022). When automated systems shape eligibility, risk scoring, prioritization, or enforcement outcomes, they participate directly in the exercise of public authority, even when human officials remain formally involved (Oswald, 2018; Civitarese Matteucci, 2021). Treating such systems as reviewable decision infrastructures allows courts to examine legality across the full administrative process rather than limiting scrutiny to final outputs.

Drawing on the doctrinal synthesis set out above, the proposed framework conceptualizes algorithmic administration as a layered structure in which authority, design, operation, and review are legally connected stages. Table 1 sets out this layered approach, identifying how reviewability, legal authority, design legality, individual fairness, and system-wide effects relate to distinct judicial questions, evidentiary needs, and remedial options. The categories in Table 1 are derived from recurring issues identified across the reviewed scholarship, official governance instruments, and illustrative judicial and administrative materials, and are presented as an analytical public law model rather than as classifications already settled uniformly across all jurisdictions. This structure responds to concerns that judicial review struggles when responsibility is dispersed across software design, data governance, and outsourced technical services (Engstrom and Ho, 2020; Gontarz, 2023). Attribution remains essential, since public bodies retain legal responsibility even where private vendors supply or maintain algorithmic tools (Cobbe and Singh, 2020; Sheehy and Ng, 2024).

Figure 1 illustrates the full decision infrastructure, showing how statutory authority flows through procurement, data selection, model configuration, operational use, and eventual review. This visualization supports a shift away from treating algorithms as background aids and toward recognizing them as sites where public law duties arise. Courts are then positioned to ask whether the system itself constrains discretion, embeds unlawful criteria, or prevents meaningful participation (Sjöberg, 2023; Mccann and Allard, 2023).

Conceptualizing algorithmic systems in this way aligns judicial review with contemporary administrative realities while preserving core public law values. Review no longer depends solely on reconstructing a single official’s reasoning but extends to examining whether the decision pathway as a whole complies with legality, fairness, and accountability standards (Harlow, 2005; Chauhan, 2020). This foundation supports later sections that specify how courts can assess design choices, evidentiary records, and remedies within algorithmic administration.

### 4.2 Legality of system design and upstream decision-making choices

Judicial review traditionally focuses on the legality of decisions as issued, yet algorithmic administration requires attention to upstream choices that shape outcomes before any individual case arises (Oswald, 2018; Haim, 2023). System objectives, data sources, thresholds, and override rules may determine how discretion is exercised, even where officials appear to apply outputs mechanically (Cobbe, 2019; Mccann and Allard, 2023). A framework that ignores these elements risks allowing unlawful criteria or improper purposes to persist through automated repetition.

Table 2 identifies a minimum review record that courts can require when reviewing algorithmic administrative systems. The items listed are synthesized from recurring documentation and oversight expectations visible in public-sector governance instruments for automated decision-making, including Canada’s guidance and impact-assessment materials, the United Kingdom’s Algorithmic Transparency Recording Standard, the EU AI Act, and the NIST AI Risk Management Framework, and are used here as doctrinally relevant benchmarks rather than jurisdiction-specific statutory mandates (Custers and Heijne, 2022; Krook et al., 2025; Wasil et al., 2025; Smuha, 2025). This review record includes lawful authority for automation, system scope, data inputs, model objectives, human oversight design, and monitoring arrangements. Each element supports established public law concerns such as relevance, fettering, fairness, and evidence disclosure duties in judicial review (Tomlinson et al., 2020; Harlow, 2005). Access to such material allows courts to test whether design choices align with statutory purposes and whether discretion has been displaced in ways that public law does not permit (Civitarese Matteucci, 2021; Williams, 2022).

Figure 2 presents a structured sequence of judicial questions that move from authority and attribution through design legality, rationality, and equality. This sequence avoids collapsing review into a single technical inquiry and instead mirrors doctrinal steps already familiar in administrative law, adapted for system-based governance (Sjöberg, 2023; Daly et al., 2023). Courts can then identify whether unlawfulness arises from data selection, model configuration, or oversight gaps rather than assuming that legality depends only on the final output.

Addressing upstream legality also responds to equality concerns, since proxy discrimination often originates in feature selection or threshold design rather than in explicit decision rules (Gerards and Zuiderveen Borgesius, 2020; Zuiderveen Borgesius, 2020). Without access to these design layers, claimants face severe barriers in showing how unequal outcomes arise (Grozdanovski, 2021; Wu, 2023). Incorporating design review into judicial scrutiny therefore strengthens both legality and equality analysis while remaining consistent with established public law principles.

### 4.3 Standards of rationality and reason-giving in automated administration

Rationality review and the duty to give reasons form core components of administrative law, yet automated systems challenge how these standards operate in practice (Williams, 2022; Cobbe, 2019). Algorithmic outputs often rely on statistical correlations, confidence thresholds, and aggregated data patterns that do not translate easily into individualized explanations (Oswald, 2018; Engstrom and Ho, 2020). Courts must therefore assess whether reasons remain intelligible and legally meaningful when decisions are shaped by encoded criteria.

Figure 3 sets out a tiered disclosure and evidence structure that distinguishes between information provided to affected persons, material available to courts, and technical documentation reviewed under protective conditions. This structure aligns rationality review with proportional disclosure, ensuring that individuals receive understandable grounds for decisions while courts retain access to deeper records needed to test legality (Coglianese and Lehr, 2019; Tomlinson et al., 2020). Rationality does not require full technical transparency for every claimant, yet it does require that courts can reconstruct how lawful considerations influenced outcomes.

Reason-giving within this framework focuses on identifying main factors, threshold effects, and decision pathways rather than reproducing model code (Sjöberg, 2023; Ranchordás, 2024). Where reasons cannot be linked to lawful purposes or where system logic obscures how criteria relate to statutory aims, rationality concerns arise. Courts may then draw adverse inferences or require further disclosure under the duty of candor (Harlow, 2005; Engstrom and Ho, 2020).

This approach preserves the normative function of reasons in administrative law, which supports participation, accountability, and review (Sela, 2016; Ng et al., 2020). Automated administration does not displace these functions, but it does require adaptation in how reasons are framed and tested. Tiered disclosure offers a workable balance that allows rationality review to operate effectively without collapsing into either technical opacity or excessive exposure of sensitive system details.

### 4.4 Procedural fairness, human oversight, and meaningful review mechanisms

Procedural fairness concerns arise sharply in automated administration because affected individuals may have limited awareness of how decisions are produced or how they can be challenged (Cobbe and Singh, 2021; Czarnocki and Pałka, 2024). Notice, opportunity to respond, and impartial reconsideration remain central requirements, yet automated workflows can weaken these safeguards when outputs are treated as definitive (Sela, 2016; Ranchordás, 2024).

Figure 4 illustrates a procedural fairness architecture that embeds notice, contest routes, data correction, and genuine human review within algorithmic decision pathways. This structure emphasizes that human involvement must include authority to reconsider outcomes and record reasons, rather than merely confirming automated results (Mccann and Allard, 2023; Cobbe, 2019). Procedural fairness fails where human review exists only in name or where time pressures and institutional incentives encourage acceptance of system outputs without scrutiny (Haim, 2023; Cobbe and Singh, 2021).

Meaningful review also depends on access to information that allows individuals to understand how data affected their case. Errors in identity, income, residence, or eligibility inputs can propagate through automated systems unless correction mechanisms are available before decisions become final (Tomlinson et al., 2020; Ranchordás, 2024). Procedural fairness therefore extends beyond hearings to include data quality controls and accessible correction routes.

Courts assessing fairness should examine whether procedural safeguards operate in practice, including whether notice is timely, explanations are comprehensible, and human reviewers have discretion to depart from system outputs (Ng et al., 2020; Czarnocki and Pałka, 2024). Where fairness mechanisms are absent or ineffective, quashing decisions or ordering procedural redesign may be appropriate. Embedding fairness into system architecture supports administrative justice while recognizing the realities of automated governance.

### 4.5 Evidence, auditability, and disclosure obligations in algorithmic decisions

Judicial review relies on evidence that allows courts to test legality, yet algorithmic administration often produces limited or fragmented records (Tomlinson et al., 2020; Engstrom and Ho, 2020). Without logs, configuration histories, or audit trails, courts face difficulty assessing how decisions were shaped or whether unlawful patterns exist across cases (Cobbe, 2019; Chauhan, 2020).

Table 3 provides an evidence and disclosure matrix that aligns categories of material with appropriate audiences and legal purposes. Claimants require access to individual factors and correction routes to support participation, while courts may require broader system documentation, performance metrics, and audit records to assess legality and equality (Coglianese and Lehr, 2019; Grozdanovski, 2021). This differentiated approach recognizes security and confidentiality concerns while maintaining effective judicial control.

Figure 5 presents a risk-tier matrix that links impact level to review intensity and evidence expectations. Higher-impact decisions affecting liberty, residence, or essential services justify deeper evidentiary access and stricter oversight (Ranchordás, 2024; Czarnocki and Pałka, 2024). Lower-impact administrative effects may justify lighter disclosure, provided basic records exist.

Auditability supports both individual justice and system-wide legality. Logs, version histories, and monitoring records allow courts to detect repeat harm, assess compliance with previous orders, and address drift over time (Engstrom and Ho, 2020; Bracci, 2023). Where evidence is missing due to agency failure, courts may draw adverse inferences or order creation of proper records. Disclosure obligations thus function as structural supports for judicial review rather than as optional administrative practices.

### 4.6 Remedies and judicial responses to unlawful algorithmic administration

Remedial design presents distinct challenges when unlawfulness arises from algorithmic systems rather than isolated administrative errors (Cromvelle, 2025; Zuiderveen Borgesius, 2020). Traditional remedies focus on individual decisions, yet automated administration can produce repeat harm across cohorts through shared design features or data inputs (Gontarz, 2023; Chauhan, 2020).

Table 4 maps types of unlawful findings to remedies that correspond to the nature and scope of harm. Individual errors may justify quashing and reconsideration, while design illegality may require declarations, redesign orders, or temporary suspension of system use (Cobbe, 2019; Williams, 2022). Evidence failures may support disclosure directions or adverse inferences, particularly where lack of records prevents effective review (Engstrom and Ho, 2020; Tomlinson et al., 2020).

Figure 6 illustrates a remedy ladder that moves from case-specific relief toward program-wide repair as harm broadens. Courts retain discretion to combine remedies, such as ordering individual relief alongside system monitoring or cohort reassessment (Cromvelle, 2025; Zuiderveen Borgesius, 2020). This flexibility supports proportional responses while avoiding judicial overreach into administrative management.

System-focused remedies also address equality concerns, since group-level metrics may reveal disparate error patterns that single-case review cannot detect (Gerards and Zuiderveen Borgesius, 2020; Weerts et al., 2023). Remedies such as targeted re-runs or disclosure of performance data support compliance with non-discrimination duties while respecting institutional limits. Judicial remedies thus remain grounded in public law principles while adapting to the realities of algorithmic governance.

### 4.7 Bounded function creep and limits on security-related repurposing

Smart city systems often collect and process data for specific administrative purposes, yet pressures to repurpose these systems for security, policing, or emergency functions raise serious legality concerns (Brauneis and Goodman, 2018; Tikhaleva, 2023). Function creep occurs when data or tools designed for one purpose are redeployed without clear legal authority or safeguards (König, 2021; Mastrogiovanni, 2025).

Table 5 provides a bounded function creep test that guides judicial assessment of repurposing decisions. This test requires courts to examine original legal bases, the nature of new uses, statutory authorization, limits on access and retention, rights impacts, and oversight arrangements (Ng et al., 2020; Zuiderveen Borgesius, 2020). Repurposing that materially alters rights exposure without explicit authority raises serious legality concerns.

Figure 7 visualizes this test as a structured decision path, supporting consistent judicial reasoning across cases. Security-related uses trigger heightened scrutiny, particularly where systems affect mobility, identity, or access to essential services (Gerards and Zuiderveen Borgesius, 2020; Czarnocki and Pałka, 2024). Courts may require suspension, new authorization, or independent oversight where safeguards are insufficient.

Bounding function creep protects public trust and preserves democratic control over data-driven governance. Judicial review plays a central role in enforcing limits, especially in contexts marked by prolonged emergency conditions or institutional fragility (Gontarz, 2023; Ranchordás, 2024). Clear tests allow courts to assess repurposing without assuming technical inevitability, ensuring that administrative power remains subject to law.

## 5 Discussion

The proposed framework carries important doctrinal meaning for common law systems, particularly because it aligns established judicial review principles with contemporary forms of administrative action shaped through automated systems. Common law doctrines have historically centered on identifiable decision-makers, written reasons, and discrete acts, yet algorithmic administration distributes decision influence across authorization, design, and operation stages (Koch, 1986; Williams, 2022). Treating algorithmic systems as reviewable decision infrastructures allows courts to preserve orthodox concepts such as legality, relevance, and fairness while adapting their application to system-based governance (Cobbe, 2019; Sjöberg, 2023). This approach does not require abandoning familiar standards of review, but it does require recognizing that unlawful discretion or improper purpose may arise earlier than the final output. The framework supports continuity with precedent while clarifying how courts can identify responsibility and assess lawfulness when administrative power is exercised through encoded processes rather than individual judgment (Harlow, 2005; Civitarese Matteucci, 2021).

Concerns about judicial capacity frequently arise in discussions of algorithmic administration, particularly regarding courts’ ability to engage with technical material (Engstrom and Ho, 2020; Tomlinson et al., 2020). The framework addresses these limits by emphasizing structured inquiry and tiered evidence rather than direct technical adjudication. Courts are not expected to assess code quality or statistical optimization, but instead to test whether lawful authority exists, whether criteria relate to statutory purposes, and whether procedural safeguards operate meaningfully (Chauhan, 2020; Sjöberg, 2023). This distinction respects institutional competence while avoiding deference that would leave automated administration beyond effective scrutiny. Judicial reliance on expert evidence, controlled disclosure, and adverse inference where records are missing further supports workable oversight (Cobbe and Singh, 2020; Engstrom and Ho, 2020). Capacity limits therefore shape how review is conducted, not whether review occurs.

The framework is especially relevant where institutional oversight is contested or fragmented and automated tools expand administrative capacity faster than review structures adapt (Gontarz, 2023; Tikhaleva, 2023). In such settings, automated tools can displace case-by-case reasoning while procedural safeguards and accessible review routes lag behind system deployment (Ng et al., 2020; Ranchordás, 2024). The framework’s focus on authority, attribution, and bounded system use provides courts with tools to assess legality even where emergency governance narratives dominate. Judicial insistence on clear authorization and effective review pathways can limit the tendency for technology-enabled measures to persist without renewed legal justification (Williams, 2022; Czarnocki and Pałka, 2024). The framework therefore supports stability in public law oversight where institutional resilience is limited.

Automated systems introduced during emergencies risk becoming permanent features of administration without renewed legal justification (Brauneis and Goodman, 2018; König, 2021). Algorithmic administration can embed emergency logics into routine decision-making, particularly where data collected for crisis response is later repurposed for control or surveillance (Mastrogiovanni, 2025; Zuiderveen Borgesius, 2020). The framework addresses this risk through its emphasis on bounded function creep and explicit authority for repurposing. Judicial review plays a key role in testing whether continued system use remains consistent with original purposes and rights protections (Gontarz, 2023; Gerards and Zuiderveen Borgesius, 2020). Without such scrutiny, automated systems may normalize heightened intrusion while reducing visibility of rights impacts. Structured review helps prevent gradual expansion of administrative power through technical means.

Governments often justify algorithmic administration on grounds of efficiency, consistency, and scale, particularly in smart city governance (Meijer and Bolívar, 2016; Coglianese and Lehr, 2019). The framework does not deny these objectives, yet it rejects the assumption that effectiveness excuses reduced legal control. Administrative effectiveness and rights protection are not mutually exclusive within public law, provided that systems are designed and governed with review in mind (Sheehy and Ng, 2024; Haim, 2023). The framework shows how judicial review can support lawful administration by identifying defects early, preventing repeat harm, and guiding corrective action (Cromvelle, 2025; Cobbe, 2019). Courts thus contribute to stable governance by ensuring that automated systems remain accountable, contestable, and legally grounded, even as administrative practices evolve through digital means.

## 6 Conclusion

This study aims to examine how judicial review can be structured to assess algorithmic administrative systems in a manner that preserves legality, fairness, and accountability within smart city governance. The analysis responded to a clear gap in the legal literature, namely the absence of a coherent public law framework that addresses algorithmic administration as a legally reviewable form of decision-making rather than a neutral technical process. Existing scholarship has examined transparency, discretion, procedural fairness, and discrimination in isolation, yet it has not provided courts with a systematic method for reviewing legality, evidence, and remedies across the full lifecycle of algorithmic systems. This study addressed that gap by developing a doctrinal framework that aligns established common law principles with the realities of automated public administration. The major conclusions of the study can be summarized as follows:

- Algorithmic administrative systems should be treated as decision infrastructures that exercise public power through authorization, design, data selection, and operational deployment, which places them squarely within the scope of judicial review under common law principles.
- Legality analysis must extend beyond individual automated outcomes to include upstream system design choices, such as objectives, thresholds, and data use, because these elements often determine how discretion and authority are exercised in practice.
- Procedural fairness in automated administration requires structured notice, contest, and human oversight arrangements that allow affected persons to understand and challenge decisions in a meaningful way, even where automation is used at scale.
- Judicial review can remain institutionally workable when courts focus on legality, relevance, and accountability rather than technical optimization, supported by tiered disclosure, expert assistance, and audit-based evidence.
- Remedies in cases of unlawful algorithmic administration must move beyond single-decision correction and include system-level responses where harm arises from repeated deployment, unequal impacts, or unlawful repurposing of civic data.

Despite these contributions, the study has limitations. The framework is developed through doctrinal and normative analysis of scholarship, selected judicial and administrative materials, and public governance instruments, rather than through empirical testing or a full comparative case-law dataset. The illustrative materials used in the study help ground the analysis, but they do not amount to jurisdiction-specific validation of each proposed category or remedy. Future research could test the framework against emerging case law, examine how courts apply evidence and remedies in practice, and assess how regulatory developments interact with judicial review in different legal systems. Further work could also examine how the framework operates in settings with limited judicial independence or administrative capacity, where algorithmic governance may pose distinct risks.

## Data Availability

The original contributions presented in the study are included in the article/supplementary material, further inquiries can be directed to the corresponding author.
